# Prognostic Value of High-sensitivity Troponin I in Patients with Septic Shock: A Prospective Observational Study

**DOI:** 10.5005/jp-journals-10071-23206

**Published:** 2019-07

**Authors:** Ali Jendoubi, Salma Jerbi, Elaa Maamar, Ahmed Abbess, Zied Samoud, Lamia Kanzari, Ilhem Boutiba, Salma Ghedira, Mohamed Houissa

**Affiliations:** 1,2,5,6,10,11 Department of Anaesthesia and Intensive Care, Charles Nicolle Hospital of Tunis, Tunisia; 3,7,8 Laboratory of Microbiology, Charles Nicolle Hospital of Tunis, Tunisia; 4,9 LR99ES09 Research Laboratory, Antimicrobial resistance, Faculty of Medicine of Tunis, University Tunis El Manar, Tunis, Tunisia

**Keywords:** High-sensitivity cardiac troponin, Mortality, Prognosis, Septic shock

## Abstract

**Background:**

Myocardial dysfunction is one of the mechanisms involved in the pathophysiology of septic shock. The role of troponin as a surrogate of myocardial injury in septic shock is still debated. The aim of this study was to assess the prognostic value of high-sensitivity cardiac troponin I (hs-cTnI) assay in predicting 28-day mortality in patients with septic shock.

**Materials and Methods:**

Prospective study including 75 patients with septic shock admitted to a medico-surgical ICU from January to December 2017. Patients under the age of 18 years, known pregnancy and patients in post–cardiac arrest were excluded. Clinical and demographic data including age, gender, comorbidities, SAPS II and SOFA scores were collected. Hs-cTnI was measured soon after admission and 12, 24, 48 and 72 after. Receiver operating characteristic (ROC) analysis was performed to identify the most useful troponin I cut-off level for the prediction of 28-day mortality. A *p* <0.05 was considered significant.

**Results:**

Seventy-five (M/F = 53/22) patients with septic shock were included in the study. The median SOFA and SAPS II scores were 10 and 42, respectively. The median duration of mechanical ventilation was 8 days and the median length of ICU stay was 11 days. The 28-day mortality was 54.6%. We found a high prevalence (47%) of elevated hs-cTnI in patients with septic shock. Median hs-cTnI on admission in the whole group was 36 ng/L. The 28-day mortality was found to be related to age (*p* <0.001), SAPS II score (*p* = 0.001), mean arterial pressure (*p* = 0.038), lactate (*p* <0.001) and glomerular filtration rate (*p* <0.001).

Hs-cTnI levels were significantly higher in non-survival group than survival one at all time points: H12 (*p* = 0.006), H24 (*p* = 0.003), H48 (*p* = 0.005) and H72 (p=0.001). In multivariate analysis, hs-cTnI at H72 was independently associated with 28-day mortality.

**Conclusion:**

Hs-cTnI elevation at 72 hours was associated with 28-day mortality in septic shock patients.

**How to cite this article:**

Jendoubi A, Jerbi S, Maamar E, Abbess A, Samoud Z, Kanzari L, *et al*. Prognostic Value of High-Sensitivity Troponin I in Patients with Septic Shock: A Prospective Observational Study. Indian J Crit Care Med 2019;23(7):320–325.

## INTRODUCTION

Septic shock is a leading cause of mortality in intensive care units (ICUs). Despite recent advances in the management of septic shock, mortality rates have remained remarkably high, ranging from 30 to 50%.^1^ Myocardial dysfunction is one of the mechanisms involved in the pathophysiology of septic shock. Septic myocardial dysfunction is usually defined as global (systolic and diastolic) but reversible biventricular dysfunction.^2^ An incidence ranging from 20 to 60% has been reported in the first 3 days after the onset of septic shock.^3^ Ventricular function generally returns to normal within 7–10 days.^4^ The pathophysiology of this dysfunction is complex and not completely understood. It involves, in addition to circulatory abnormalities, alterations in coronary blood flow, circulating depressant factors, microvascular dysfunction, abnormalities of beta-adrenergic signal transduction^5^, apoptotic phenomena and calcium dysregulation.^6^ Cardiac troponins are specific biomarkers of myocardial cell injury and their role for risk stratification in acute coronary syndromes is well established.^7^ The role of troponin in risk stratification of sepsis is still debated.^8,9^ A new generation of highly sensitive troponin assays has recently been developed that allow the detection of concentrations 10 times lower than those measureable with conventional assays.^10^ The aim of this study was to determine the association between high-sensitivity cardiac troponin I (hs-cTnI) elevation and 28-day mortality in patients with septic shock.

## MATERIALS AND METHODS

After approval of the Local Ethics Committee, a single center prospective observational cohort study was conducted in a 12-bed mixed surgical and medical ICU over a 12-month period, from 1st January 2017 to 31st December 2017. This study included consecutive septic shock adult patients (18 years old or more) admitted to the ICU. Septic shock was defined as sepsis with hypotension despite initial volume resuscitation of 30 mL/kg body weight in accordance with the American College of Chest physicians (ACCP)/Society of Critical Care Medicine (SCCM) Consensus Conference Committee.^11^ Patients under the age of 18 years, known pregnancy and patients in post–cardiac arrest were excluded.

Baseline clinical variables including age, gender, body mass index (BMI), cause of sepsis and preexisting comorbidities were collected. The severity of disease was assessed by SAPS II (Simplified Acute Physiology Score) and the SOFA (Sepsis-related Organ Failure Assessment) scores. At ICU admission, clinical and biological parameters including heart rate (HR), mean arterial pressure (MAP), complete blood count, C-reactive protein (CRP), serum creatinine, estimated glomerular filtration rate (eGFR) using the Modification of Diet in Renal Disease MDRD formula, total bilirubin, albumin, lactate, prothrombin time (International Normalized Ratio) and arterial blood gas analysis were also collected. Lengths of mechanical ventilation and ICU stay were recorded, ICU and 28-day mortality were assessed. Patients were subjected to transthoracic echocardiography (TTE) *at* study inclusion and cardiac output measurement was performed.

*Biochemical analysis:* We measured troponin concentrations in plasma soon after admission in ICU and 12, 24, 48 and 72 hours after; using a high-sensitivity troponin I assay (Abbott ARCHITECT STAT, Abbott Laboratories). The recommended cut-off value for an elevated cardiac troponine is the 99^th^ percentile of a control reference group at a precision level ≤10% coefficient of variation. The high-sensitivity assay is reported in units of ng/L. The 99^th^ percentile upper reference limit (URL) for this test is 34 ng/L for men and 16 ng/L for women. The limit of detection is 1.9 ng/L and the interassay coefficient *of* variation is <10% at 4.7 according to the manufacturer's specification.^10^

*Outcomes:* The primary study outcome was 28-day mortality. Predefined secondary outcomes were: duration of mechanical ventilation and length of ICU and hospital stay.

*Statistical analysis:* Continuous variables are expressed as mean ± standard deviation or median (interquartile range). Categorical variables were compared using χ^2^ analysis, and continuous variables with normal distributions were compared using the Student's *t* test. The Mann–Whitney U test was used to compare continuous variables with a skewed distribution. Discrimination between hospital survivors and non-survivors was evaluated by receiver operating characteristic (ROC) curve analysis.

Variables with a *p* value < 0.02 on bivariate analysis were included in the logistic regression model for multivariate analysis of 28-day mortality. Adjusted ORs were calculated using a logistic regression model. All statistical analyses were performed using the Statistical Package for the Social Sciences 18.0 software (SPSS Inc., Chicago, IL, USA). *p* values less than 0.05 were considered statistically significant.

## RESULTS

Seventy-five patients with septic shock were included in the study. **Table 1** shows the baseline characteristics of the entire cohort. Thirty five (47%) patients had elevated troponin I levels. The 28-day mortality was 54.6% (41/75).

Characteristics of the patients according to 28-day mortality are summarized in **Table 2**. Survivors were significantly younger than non-survivors and had significantly lower severity of illness (SAPS II score). In the univariate analysis, 28-day mortality was found to be related to low MAP (*p =* 0.038) and high lactate level (*p* <0.001). The eGFR was significantly lower in non-survivors than in survivors (*p* <0.001). Baseline median hs-cTnI levels were not significantly higher in non- survivors than survivors (*p* = 0.126) but the serial measurements over a 72-hour period showed that hs-cTnI levels were significantly higher in non*-*survival group than survival group at all time points **H12** (*p* = 0.003), **H48** (*p* = 0.005) and **H72** (*p* = 0.001).

**Table 1:** Baseline characteristics of the study population

*Variables*	*Values (n = 75)*
Age (years) median (min-max)	57(18-93)
Male gender n (%)	53 (70%)
SAPSII median [IQR]	42 [34 – 53]
SOFA median [IQR]	10 [8 – 11]
*Comorbidities*	
Diabetes mellitus n (%)	22/75 (29.3%)
Hypertension n (%)	16/75 (21.3%)
Atrial fibrillation n (%)	8/75 (10.7%)
Dyslipidemia n (%)	4/75 (5.4%)
*Laboratory tests and ABG analysis*	
CRP (mg/L) mean ± SD	194.5 ± 113.8
Creatinine clearance MDRD (mL/min) mean ± SD	87.1 ± 50.9
Leucocytes (x10^9^/L) mean ± SD	18.02 ± 7.99
Platelet count (x10^9^/L) mean ± SD	222.11± 144.51
PaO_2_/FiO_2_ mean ± SD	248.47 ± 15.97
*Macro- and microcirculatory variables*	
HR (bpm) mean ± SD	107.08 ± 24.53
MAP (mm Hg) mean ± SD	78.81 ± 11.88
Cardiac index l/min/m^2^ mean ± SD	2.48 ± 0.77
Blood Lactate level (mmol/L) median [IQR]	1.9 [1.1 – 3.2]
*High-sensitivity cardiac troponin I concentrations*	
Hs-cTnI H0 ng/L median [IQR]	36 [12 – 108]
Hs-cTnI H12 ng/L median [IQR]	43 [13 – 342]
hs-cTnI H24 ng/L median [IQR]	42 [18 – 253]
hs-cTnI H48 ng/L median [IQR]	51 [10 – 124]
hs-cTnI H72 ng/L median [IQR]	21 [9 – 94]
*Norepinephrine µg/kg/min median [IQR]*	*0.44 [0.22 – 0.69]*
*Outcome variables*	
Length of ICU stay days median [range]	11 [5 – 20]
Duration of Mechanical Ventilation days median [IQR]	8 [3 – 14]
28-day mortality n (%)	41/75 (54.6%)

Data are presented as means ± standard deviation (SD), medians (Interquartile range Q1–Q3) or absolute numbers (percentage). SAPS II, Simplified Acute Physiology Score. SOFA: Sequential Organ Failure Assessment; MAP, Mean arterial pressure; HR, Heart rate; ABG, Arterial Blood Gazs.

Our patients were then categorized into 2 groups: hs-cTnI (+) positive group (serum hs-cTnI level, M: ≥34 ng/L; F: ≥16 ng/L) and hs-cTnI (–) negative group (serum hs-cTnI level, M: <34 ng/*L*; F: <16 ng/L). Baseline characteristics of the cohorts with and without hs-cTnI elevation are detailed in **Table 3**. There were no significant differences between two groups at baseline regarding the age, MAP, cardiac index, vasopressor or ventilatory requirements and lactate levels. The eGFR was significantly lower in the hs-cTnI + positive group (*p* = 0.017).

**Fig. 1:**
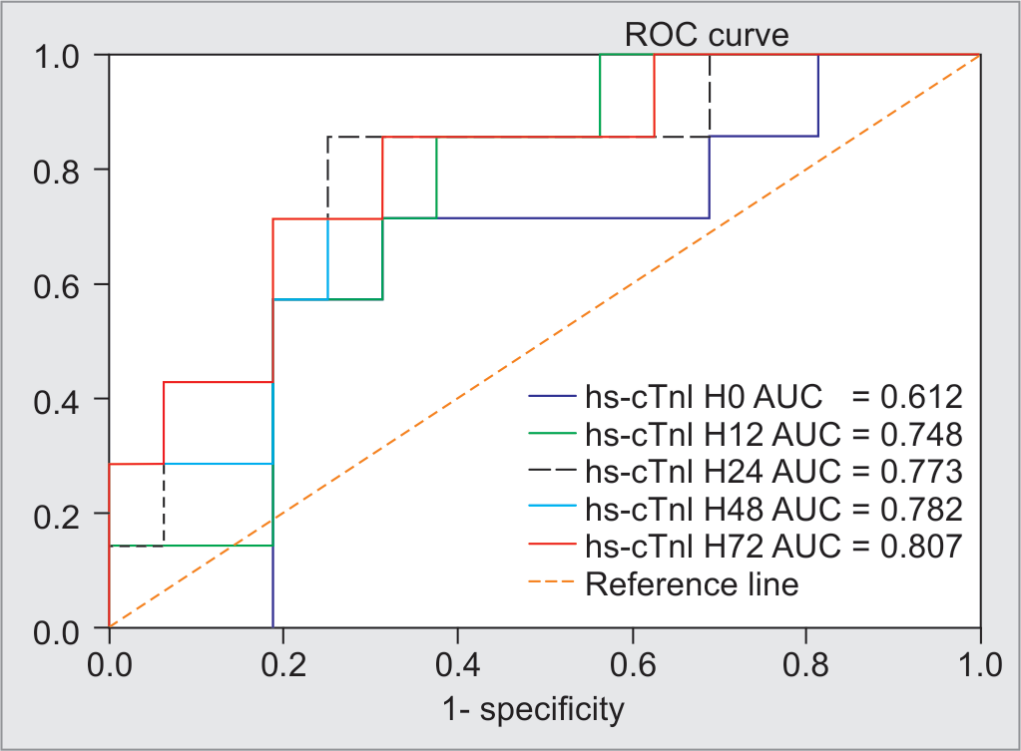
Comparison of ROC curves between hs-cTnI values at different time points

The prediction of mortality was assessed using the area under the ROC curve (AUC). The AUC for hs-cTnI at admission was 0.61 [95% CI, 0.47–0.75; *p* = 0.126] **(Fig. 1)**. The AUC value of 0.61 suggests the admission troponin level is poorly discriminatory when used to predict the 28-day mortality among patients with septic shock. We also compared the discriminatory power between hs-cTnI values at different time points (H12, H24, H48 and H72) for predicting 28-day mortality. The best cut-off values for troponin levels for mortality, obtained from ROC curves **(Fig. 1)**, are shown in **Table 4**. A troponin level greater than 17 ng/l at H72 was the strongest predictor of 28-day mortality, with an odds ratio of 8.5.

**Table 2:** Characteristics of the study patients according to 28-day survival status

*Variables*	*Non-survivors* *(n = 41)*	*Survivors* *(n = 34)*	*p value*
Age (years) median (min-max)	60(26-93)	40(18-90)	**<0.001**
SAPSII median [IQR]	49 [37 – 63]	36 [29 – 45]	**0.001**
SOFA median [IQR]	10 [8 – 12]	10 [8 – 11]	0.213
*Laboratory tests and ABG analysis*			
CRP (mg/L) median [IQR]	218 [114 – 285]	166 [86 – 259]	0.213
Creatinine clearance MDRD (mL/min) median [IQR]	54 [29 – 106]	110 [85 – 135]	**<0.001**
Leucocytes (x10^9^/L) mean ± SD	19.07 ± 8.50	16.44 ± 7.22	0.188
Platelet count (x10^9^/L) mean ± SD	225.32± 156.21	216.87± 136.65	0.907
PaO_2_/FiO_2_ median [IQR]	220 [139 – 308]	233 [176 – 384]	0.333
*Macro- and microcirculatory variables*			
HR (bpm) median [IQR]	111 [92 – 128]	102 [83 – 113]	0.076
MAP (mmHg) median [IQR]	75 [69 – 85]	84 [73 – 90]	**0.038**
Cardiac index l/min/m^2^ median [IQR]	1.9 [1.4 – 3.1]	2.5 [1.9 – 3.1]	0.179
Blood Lactate level (mmol/L) median [IQR]	2.5 [1.7 – 3.7]	1.1 [0.8 – 1.9]	**<0.001**
Norepinephrine µg/kg/min median [IQR]	0.53 [0.18 – 0.88]	0.43 [0.22 – 0.62]	0.211
*High-sensitivity cardiac troponin I*			
Hs-cTnI H0 ng/L median [IQR]	57 [12 – 115]	23 [10 – 54]	0.126
Hs-cTnI H12 ng/L median [IQR]	133 [40 – 576]	22 [8 – 176]	**0.006**
hs-cTnI H24 ng/L median [IQR]	101 [34 – 428]	23 [8 – 121]	**0.003**
hs-cTnI H48 ng/L median [IQR]	84 [46 – 351]	17 [8 – 81]	**0.005**
hs-cTnI H72 ng/L median [IQR]	76 [21 – 414]	11 [6 – 27]	**0.001**

Data are presented as means ± standard deviation (SD), medians (Interquartile range Q1–Q3) or absolute numbers (percentage); SAPS II, Simplified Acute Physiology Score; SOFA, Sequential Organ Failure Assessment; MAP, Mean arterial pressure; HR, Heart rate; ABG, Arterial Blood Gaz.

**Table 3:** Mortality and baseline characteristics of the study population by baseline high-sensitivity troponin T levels

*Variables*	*hs-cTnI* ^*+*^ *group* *(n = 40)*	*hs-cTnI* ^*-*^ *group* *(n = 35)*	*p value*
28-day mortality (NS/S)	25/15	16/19	0.070
Age (years) median (min-max)	57(18-93)	57(25-90)	0.838
SAPSII median [IQR]	45 [34 – 55]	38 [29 – 50]	0.136
SOFA median [IQR]	10 [8 – 12]	10 [8 – 11]	0.825
CRP (mg/L) median [IQR]	207[100 – 269]	197 [106 – 277]	0.942
eGFR MDRD (mL/min) median [IQR]	54 [33 – 107]	99 [51 – 135]	**0.017**
Leucocytes (x10^9^/L) mean ± SD	19.11 ± 8.30	16.91 ± 7.34	0.164
Platelet count (x10^9^/L) mean ± SD	226.28± 141.42	184.33± 99.77	0.201
PaO_2_/FiO_2_ median [IQR]	243 [147 – 355]	234 [160 – 314]	0.778
HR (bpm) median [IQR]	110 [92 – 125]	102 [89 – 120]	0.257
MAP (mm Hg) median [IQR]	80 [70 – 88]	81 [72 – 87]	0.585
Cardiac index l/min/m^2^ median [IQR]	1.9 [1.4 – 3.1]	2.5 [1.9 – 3.1]	0.179
Blood lactate level (mmol/L) median [IQR]	2.5 [1.9 – 3.1]	2.4 [1.9 – 2.9]	0.683
Norepinephrine µg/kg/min median [IQR]	0.44 [0.19 – 0.79]	0.45 [0.27 – 0.66]	0.696

Based on the data of all the patients, the following variables were selected (P ≤0.02) by the stepwise logistic regression procedure: age, SAPS II score, admission lactate level, creatinine clearance at ICU admission, and troponin level at H72.

**Table 4:** Best cutoff values of troponin that were obtained from ROC curves for mortality

	*AUC (CI 95%)*	*Cutoff (ng/l)*	*OR*	*Se (%)*	*Sp (%)*	*p*
hs-cTnI H12	0.748 (0.548-0.948)	50	5.8	70	76	0.061
hs-cTnI H24	0.773 (0.566-0.980)	30	5.3	80	62	0.039
hs-cTnI H48	0.782 (0.583-0.980)	40	6.4	82	65	0.033
hs-cTnI H72	0.807 (0.614-0.999)	17	8.5	63	81	0.020

Hs-cTnI, high-sensitivity cardiac troponin I; AUC, area under the ROC curve

When adjusting for these significant variables, the P value for hs-cTnI at H72 was 0.001 (OR 16.8; 95% CI, 3.417-82.603), demonstrating that troponin level was an independent prognosticator **(Table 5).**

## DISCUSSION

In the present study, we found a high prevalence (47%) of elevated hs-cTnI in patients with septic shock. The serial measurements of cardiac troponins over a 72-hour period showed that hs-cTnI levels were significantly higher in non-survival group than survival group at all time points H12, H24, H48 and H72. In multivariate analysis, hs-cTnI at H72 was independently associated with 28-day mortality, with a hazard ratio of 16.8 (95% confidence interval [CI] 3.417-82.603; *p* = 0.001).

Recently, several studies have also reported detectable troponin levels in septic patients, but their predictive value has not been firmly established. The reasons for circulating cardiac troponins elevation in severe sepsis and septic shock are still debated. Troponin release in sepsis does not necessarily indicate cardiomyocyte necrosis, but could also be a result of increased cell permeability and the release of troponin degradation products through the cell membrane in non-necrotic cardiomyocytes.^12,13^

**Table 5:** Multivariable logistic regression analysis of variables for mortality

*Variable*	*OR*	*95% Confidence interval*		*p*
		*Lower*	*Upper*	
Age	1.04	0.991	1.081	0.119
SAPS II	0.20	0.019	2.112	0.182
Lactate	3.82	0.678	21.530	0.128
eGFR	0.99	0.975	1.017	0.690
Hs-cTnI at H72	16.80	3.417	82.603	**0.001**

SAPS II, standardized *index* of gravity; OR, Odds Ratio; Hs-cTnI, high-sensitivity cardiac troponin I; eGFR, estimated glomerular filtration rate

A close association between hs-cTnT and NT-proBNP levels in septic shock was reported in the ALBIOS Trial^14^ highlighting the value of troponin in simultaneous assessment of myocardial function and circulatory status. In a recent study by Landesberg and colleagues,^15^ left ventricular diastolic dysfunction and right ventricular dilatation were found to be strongly associated with hs-cTnT positivity and mortality. Ver Elst et al.^16^ noted that 78% of cTnI+ patients had reduced left ventricular ejection fraction compared with 9% of cTnI− individuals. Fernandes et al.^17^ observed similar findings. No differences in the coagulation parameters analyzed with rotational thrombelastometry were found between cTnI positive and negative patients with systemic inflammatory response syndrome (SIRS), sepsis or septic shock.^18^

Several pathophysiological mechanisms other than thrombus-associated myocardial damage could play a major role, including reversible myocardial membrane leakage and/or cytokine mediated apoptosis in these patients. It has been suggested that sepsis-induced myocardial dysfunction involves pro-inflammatory cytokines including tumour necrosis factor-α (TNF-α), interleukin-1β (IL-1β) and interleukin-6,^19^ nitrite oxide, alteration of beta-adrenergic receptors,^20^ apoptosis and calcium abnormalities particularly a decreased myocardial fibers sensibility.^5^

In our study, the prevalence of increased cardiac troponin I levels was 47%. In a heterogeneous population of patients with sepsis, severe sepsis, and septic shock, the incidence of elevated troponin ranged from 55% to 85%.^12,17,21–23^ In studies involving patients with septic shock, there was an incidence of elevated cardiac troponin I ranging from 43% to 80%.^16,24,25^ In a meta-analysis involving 13 studies and 1227 patients with sepsis, Bessière and colleagues reported a prevalence of 61% of elevated troponin in patients.^8^

There are only a few studies in the literature that have reported increased levels of hs-cTnI in patients with septic shock. In a post hoc analysis of a cohort of 995 patients with severe sepsis or septic shock participating in the ALBIOS trial, Masson S. et al.^14^ showed high levels of hs-cTnT in up to 84.5% of septic shock patients. In this predefined substudy, the hs-cTnT levels were twice as high in patients with shock as compared to those with severe sepsis without shock. In a recent study published in February 2018 by Frencken et al.;^26^ the researchers performed daily measurements of hs-cTnI in a large cohort of 1256 patients hospitalized in ICU with sepsis. The authors found that 60% of the subjects had a troponin level above the upper reference limit of the test (26 ng/L) on day 1, and an additional 82 (7%) developed raised concentrations within the first 4 days in the ICU.

The main finding in our study is the close association between increased hs-cTnI concentrations at 72 hours and 28-day mortality. Prior studies on sepsis and septic shock have presented conflicting data on the association of clinical outcomes with troponin elevation.

*Positive studies:* troponin levels were independently associated with mortality after sepsis in 2 recent meta-analyses.^8,9^ In a large retrospective study of 598 patients, John J. et al.^21^ found that elevated cTnI was an independent prognosticator of 28-day mortality in severe sepsis patients (OR: 2.02; 95% CI: 1.15-3.54; *p* <0.05). In another retrospective cohort study conducted in Mayo Clinic, USA, and involving 944 patients with severe sepsis and septic shock, the authors found that elevated admission troponin-T was associated with higher short- and long term mortality (in-hospital mortality : OR 1.4; *p* = 0.04; 1-year mortality: OR 1.3; *p* = 0.008).^27^ Previous studies^15,21,25,28^ have demonstrated that myocardial injury is predictive of in hospital or 28-day mortality in patients with severe sepsis or septic shock. In a meta-analysis of 17 studies with total sample size of 1857 patients, the authors found that elevated troponin was significantly associated with mortality (Risk ratio: 1.91; 95% CI: 1.65-2.22; *p* <0.05).^9^ Recent findings from a large scale prospective cohort study in the Netherlands (n= 1124 septic patients), Frencken et al.^26^ found that hs-cTnI concentrations were elevated in 673 (60%) subjects on day 1, and 755 (67%) ever had elevated levels in the first 4 days.

*Negative studies:* In contrast, several studies have not found elevated troponin to be an independent predictor of mortality in septic patients.^29,30^ In a study of a cohort of 159 patients with bacteraemia reported that cTnI measured with a conventional assay is a univariate, but not an independent predictor of outcome.^31^ In a subgroup of 207 patients from FINNSEPSIS trial^32^ (a large prospective observational cohort study of the incidence and prognosis of sepsis in 24 intensive care units (ICUs) in Finland),^33^ the level of hs-cTnT on inclusion was higher in hospital non-survivors than survivors (*p* = 0.047), but hs-cTnT level was not an independent predictor of in-hospital mortality.

*Study limitations:* Several limitations in our study need to be mentioned: The first is that we conducted a single centre study. Second, the generalisability of our findings is limited by the small sample size. Third, and most importantly, we have not reported electrocardiographic changes and have not assessed the cardiac function by echocardiography (evaluation of the left and right ventricular systolic and diastolic function).

*Implications for practice:* Our results suggest questions regarding the role of high-sensitive cardiac troponin I assays during septic shock management and highlight the need for further studies to identify which subgroup of septic patients needs serial troponin testing and eventually requires aggressive management of shock and to evaluate prognostic value of hs-cTnI for long-term outcomes including all-cause mortality and major cardiovascular events at long*-*term (>1 year) follow-up. The application of this biomarker-based approach could help stratify risk in the emergency department.
